# Improved selection of zebrafish CRISPR editing by early next-generation sequencing based genotyping

**DOI:** 10.1038/s41598-023-27503-9

**Published:** 2023-01-27

**Authors:** Ewa Sieliwonczyk, Bert Vandendriessche, Charlotte Claes, Evy Mayeur, Maaike Alaerts, Philip Holmgren, Tycho Canter Cremers, Dirk Snyders, Bart Loeys, Dorien Schepers

**Affiliations:** 1 Faculty of Medicine and Health Sciences, Center for Medical Genetics, University of Antwerp and Antwerp University Hospital, Antwerp, Belgium; 2 Experimental Neurobiology Unit, Department of Biomedical Sciences, University of Antwerp, Antwerp, Belgium; 3 Department of Clinical Genetics, Radboud University Medical Center, Nijmegen, The Netherlands

**Keywords:** Zebrafish, Transgenic organisms, Animal disease models, Genetic engineering

## Abstract

Despite numerous prior attempts to improve knock-in (KI) efficiency, the introduction of precise base pair substitutions by the CRISPR-Cas9 technique in zebrafish remains challenging. In our efforts to generate KI zebrafish models of human *CACNA1C* mutations, we have tested the effect of several CRISPR determinants on KI efficiency across two sites in a single gene and developed a novel method for early selection to ameliorate KI efficiency. We identified optimal KI conditions for Cas9 protein and non-target asymmetric PAM-distal single stranded deoxynucleotide repair templates at both *cacna1c* sites. An effect of distance to the cut site on the KI efficiency was only observed for a single repair template conformation at one of the two sites. By combining minimally invasive early genotyping with the zebrafish embryo genotyper (ZEG) device and next-generation sequencing, we were able to obtain an almost 17-fold increase in somatic editing efficiency. The added benefit of the early selection procedure was particularly evident for alleles with lower somatic editing efficiencies. We further explored the potential of the ZEG selection procedure for the improvement of germline transmission by demonstrating germline transmission events in three groups of pre-selected embryos.

## Introduction

With recent advances in gene editing, the creation of in vivo genetic models of human disease has steadily become more feasible^1^. Zebrafish have proven an especially attractive genetic animal model, as they are generally easier to genetically modify compared to other vertebrates^2–4^. The use of CRISPR-Cas9 has greatly expedited the generation of genetic knock-out (KO) models by inducing out-of-frame insertions or deletions (indels) in genes of interest via the non-homologous end joining (NHEJ) repair pathway^5,6^. However, KO modelling provides only a limited representation of the spectrum of human genetic diseases, which are more often caused by missense mutations based on single base pair (BP) substitutions, rather than indels^7^. Complete KO models are unable to accurately represent the molecular consequences of single BP substitutions, such as gain-of-function, dominant negative effects or alterations in protein trafficking or processing pathways. The frequent identification of genetic variants of uncertain significance in the clinic^8^ further drives the increased demand for functional tools for the assessment of specific mutations to aid in variant classification^9^.

Single BP substitutions can also be generated by CRISPR-Cas9 via the repair pathway of homology-directed repair (HDR)^5^. These knock-in (KI) models provide the opportunity to study the effect of specific mutations. However, as HDR occurs less frequently than NHEJ, KI models are more difficult to generate^10,11^. The improvement of this process is an active field of research, with aims to increase the KI rate in zebrafish by, predominantly, modifications of the CRISPR-Cas9 components^10,12–14^ and the addition of compounds which affect the repair pathway^10,15^.

Despite these efforts, the identification of the optimal KI conditions for zebrafish is hampered by locus specific differences in CRISPR-Cas9 function as well as variable methodologies to determine the KI efficiency. Since it is often unclear whether the identified improvements depend on the (epi)genomic environment of the mutation^13^, it can be difficult to extrapolate experimental findings to other loci. Different methods for the estimation of KI rates in injected embryos (e.g. allele-specific PCR^12^ or sequencing methods^16^), render the comparison between different experiments even more challenging. In practice, typical KI rates are estimated at 1–4%^10^, which makes the generation of KI lines a laborious process.

In this paper we examine the contribution of several CRISPR determinants (single guide RNA (sgRNA), cutting efficiency, use of Cas9 protein or mRNA, repair template conformation and distance of the intended mutation from the sgRNA cut site) on the generation of a KI model. We focus on the editing of two sites within a single gene, and thus provide an opportunity to examine similarities between the conditions at these loci and identify potential gene or locus specific effects. For this purpose, we chose to model known human pathogenic variants in the *CACNA1C* gene, encoding a cardiac calcium channel. Exploration of the phenotype of zebrafish *cacna1c* mutants will provide a valuable contribution to cardiovascular research as zebrafish KI models of *cacna1c* mutations have not been previously generated. In addition, due to the frequent identification of variants of uncertain significance in *CACNA1C* in the clinic, there is a need for a novel functional assay to evaluate the pathogenicity of genetic variants in this gene. This would be facilitated by improving the generation process of *cacna1c* KIs.

Additionally, we investigate whether the combination of early genotyping with a next-generation sequencing (NGS) based KI detection method can help facilitate the generation of KI models. We make use of the Zebrafish Embryo Genotyper (ZEG) device, which can extract a small amount of genomic DNA from 72 h post-fertilization embryos, with minimal lethality^17,18^. These DNA samples are analyzed by NGS to identify zebrafish embryos with the highest rate of correctly edited cells, and selectively raise these embryos to adulthood. By pre-selecting embryos with higher KI rates, we intend to reduce cost, time and number of laboratory animals required to achieve germline transmission.

## Results

### Comparison of indel frequency

For our CRISPR-Cas9 experiments, we focused on two known mutations in the *CACNA1C* gene: c.2570C>G or p.(Pro857Arg), corresponding to c.2607C>G or p.(Pro871Arg) in zebrafish, associated with a long QT syndrome (LQTS) phenotype^19^ and c.989C>T or p.(Thr330Met), corresponding to c.1028C>T or p.(Thr343Met) in zebrafish, associated with Brugada syndrome (BrS)^20^. As successful KI generation is highly reliant on a sgRNA with a high cleavage capacity, we first sought to identify the optimal method for sgRNA selection by comparing different techniques for indel detection. Although a precise indel estimation can also be obtained with NGS, other methods are more cost-effective.

**Figure 1:**
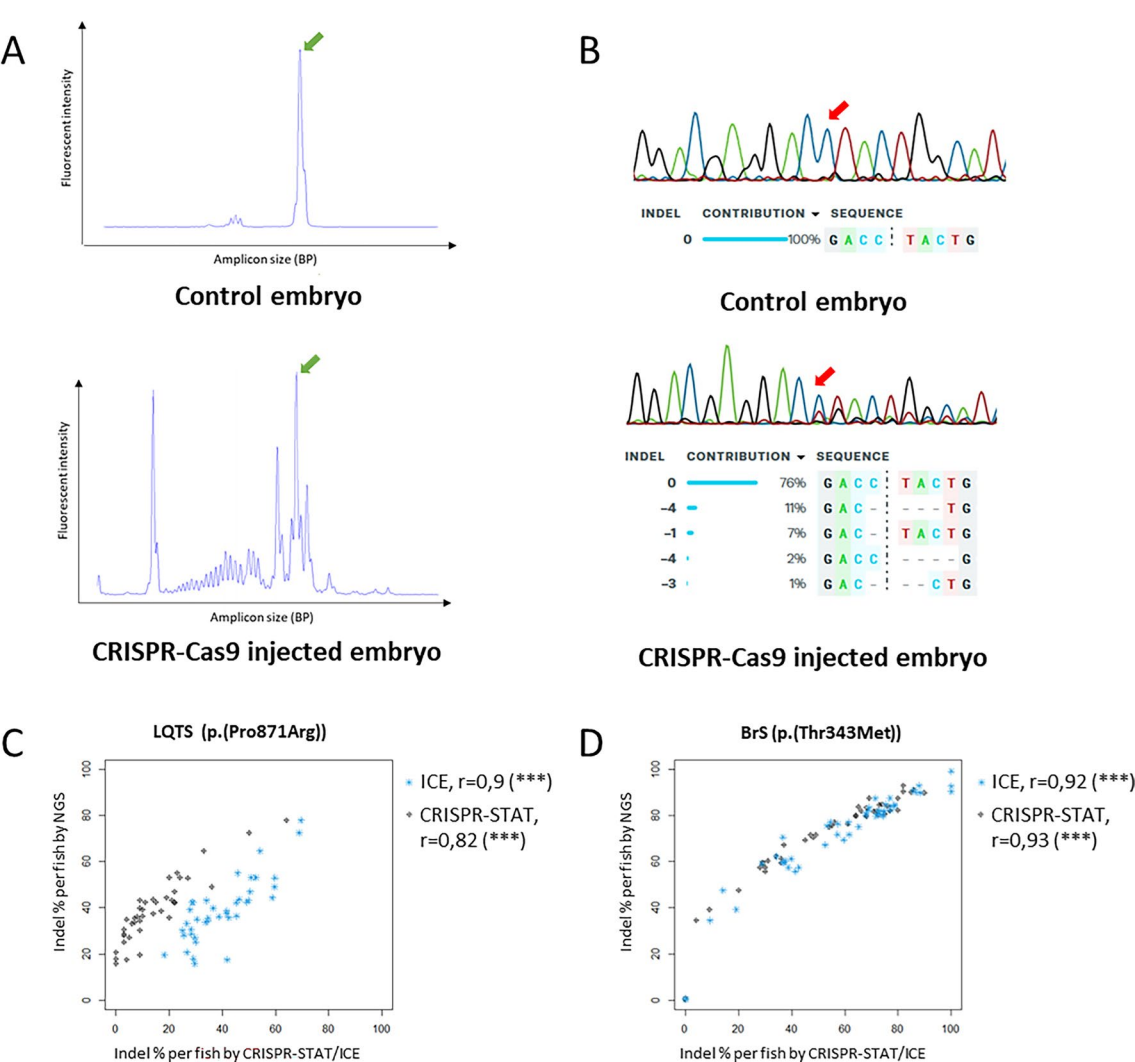
Comparison of indel detection methods. (**A**) Representative traces for CRISPR-STAT, top: sample from uninjected control embryo, bottom: sample from embryo injected with CRISPR-Cas9 components, green arrow: peak from wildtype amplicon, all other peaks in injected embryo represent different indel events. (**B**) Representative Sanger chromatograms and ICE analysis results, top: sample from uninjected control embryo, bottom: sample from embryo injected with CRISPR-Cas9 components, the trace from the injected embryo shows additional peaks on the right side of the cut site (red arrow), representing different indels, which are detected by ICE. (**C,D**) Correlation between Inference of CRISPR Edits (ICE) (blue symbols), CRISPR-STAT (black symbols) and NGS for LQTS (**C**) and BrS locus (**D**). Each symbol represents the indel percentage calculated for the individual embryo. *Indel* insertions/deletions, *NGS* next generation sequencing, *BP* base pair.

For this purpose, we calculated the indel percentage for individual embryos injected with sgRNA and Cas9 protein or mRNA based on CRISPR-STAT and Inference of CRISPR Edits (ICE) methods (Fig. 1A,B) and compared these values to the data obtained by NGS (Fig. 1C,D). For the LQTS locus, we observed a significant correlation for CRISPR-STAT (Pearson’s r = 0.82, p < 0.001, n = 41) and ICE (Pearson’s r = 0.90, p ≤ 0.001, n = 41) (Fig. 1C). Similarly, at the BrS locus, a significant correlation was observed for both CRISPR-STAT (Pearson’s r = 0.93, p < 0.001, n = 49) and ICE (Pearson’s r = 0.92, p < 0.001, n = 49) (Fig. 1D). A more detailed analysis of the indel profiles revealed that very small (1–2 BP) indels were often missed by CRISPR-STAT, as the peaks were difficult to distinguish from wildtype. The higher incidence of small (1 BP) indels at the LQTS locus may partly explain the lower correlation with the NGS data, compared to the BrS locus (Supplementary Tables S1, S2). Both the ICE and CRISPR-STAT methods appeared to underestimate the cutting efficiency, especially at lower percentages. Overall, we concluded that while both methods are appropriate for sgRNA comparison, ICE provides more objective results, performs faster, and leads to fewer errors in the estimation of small indels.

### Contribution of CRISPR-Cas9 components to the KI efficiency

**Figure 2:**
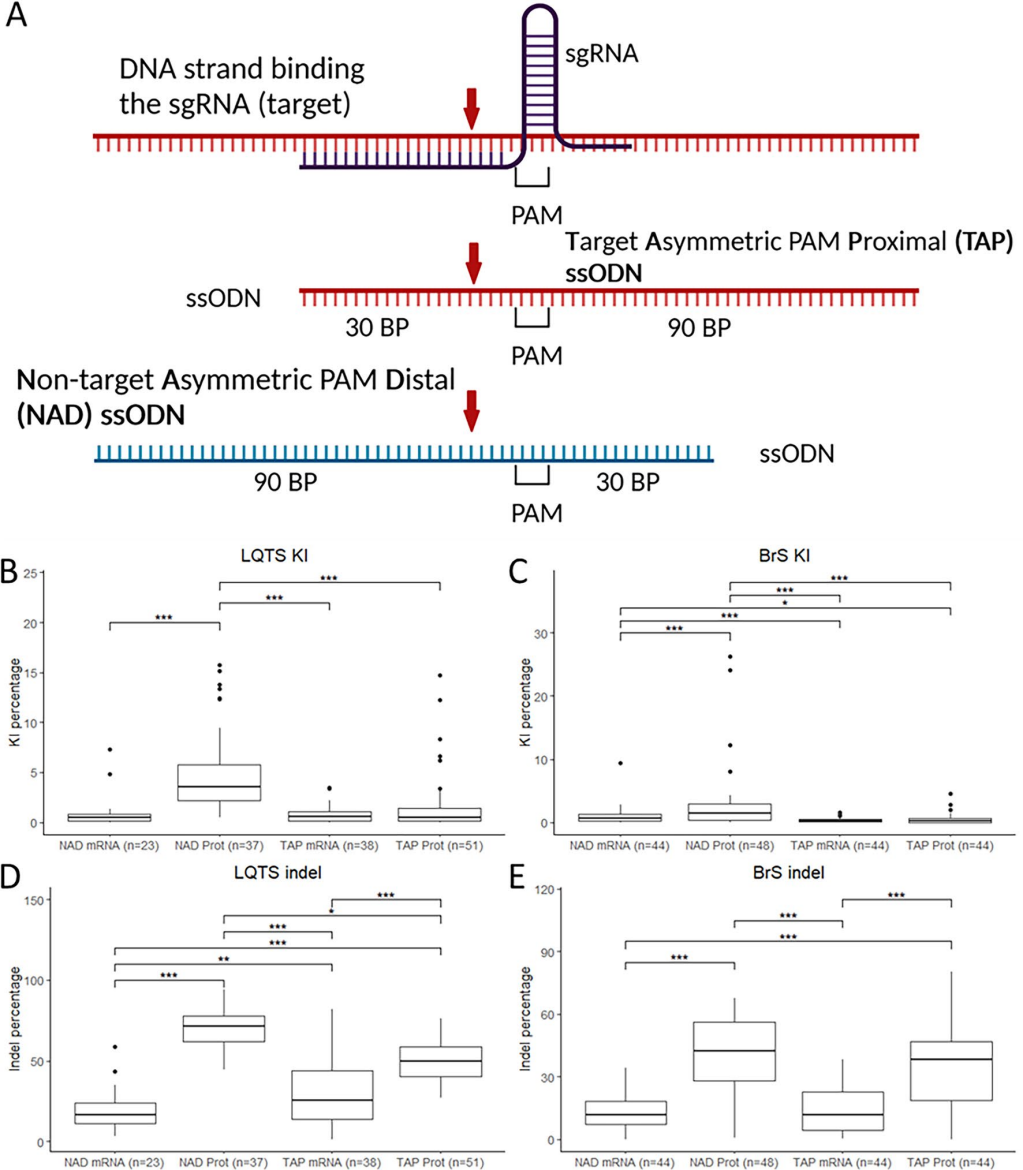
Editing efficiency with different injection components. (**A**) ssODN conformations used for the experiments, red arrow: CRISPR-Cas9 cut site. (**B,C**) KI efficiencies with the different ssODN conformations and Cas9 protein/mRNA for the LQTS locus (**B**) and the BrS locus (**C**). (**D,E**) Indel percentages with the different ssODN conformations and Cas9 protein/mRNA for the LQTS locus (**D**) and the BrS locus (**E**). Statistical testing was performed with negative binomial regression with use of the Tukey’s HSD (honestly significant difference) test for multiple correction, not significant results are not displayed, ***p-value < 0.001, **p-value < 0.01, *p-value < 0.05. *PAM* protospacer-adjacent motif, *BP* base pairs, *NAD* non-target asymmetric PAM distal, *TAP* target asymmetric PAM proximal, *KI* knock-in, *indel* insertion/deletion.

As sgRNAs at both loci were able to successfully induce double stranded DNA breaks, we sought to characterize other injection components which might affect KI efficiency in order to identify the optimal editing conditions for the *cacna1c* gene. We examined the effect of two injection components: Cas9 mRNA or protein, as well as two single stranded deoxynucleotide (ssODN) conformations (target asymmetric PAM-proximal (TAP) and non-target asymmetric PAM-distal (NAD)), with a length of 120 BP (Fig. 2A, Supplementary Fig. S1) based on negative binomial regression^10^. In case of the PAM-proximal conformation, the long arm contains the PAM site, while for the PAM-distal conformation, the PAM site is located on the short arm. These conformations were selected based on their superior performance demonstrated in Boel et al.^10^.

The NAD conformation with Cas9 protein significantly outperformed all other conditions for both loci, with an average somatic editing efficiency of 5.14% ± 0.71 for the LQTS locus (p-value < 0.001, Fig. 2B) and 2.83% ± 0.75 for the BrS locus (p-value < 0.001, Fig. 2C). At both loci, the use of Cas9 protein led to significantly higher editing frequencies for the NAD conformation compared to Cas9 mRNA, with an average somatic editing frequency of 5.14% ± 0.71 for protein and 0.94% ± 0.35 for mRNA at the LQTS locus (p-value < 0.001, Fig. 2B) and 2.83% ± 0.75 for protein and 1.04% ± 0.22 for mRNA at the BrS locus (p-value < 0.001, Fig. 2C). We observed a similar trend towards an increased performance with Cas9 protein for the TAP conformation with and average editing rate of 1.51% ± 0.40 for protein and 0.78% ± 0.14 for mRNA at the LQTS locus (p-value 0.0573, Fig. 2B) and 0.52% ± 0.13 for protein and 0.32% ± 0.05 for mRNA at the BrS locus (p-value 0.322, Fig. 2C).

Additionally, Cas9 protein injections led to significantly higher indel percentages compared to mRNA for both ssODN conformations at both loci (Fig. 2D,E). At the LQTS locus, an increased indel percentage was observed for the NAD conformation (69.69% ± 1.83) compared to TAP (50.5% ± 1.84, p-value 0.004, Fig. 2D) based on Cas9 protein. This was likely due to variability in injection efficiency that may have contributed to the high somatic editing efficiency observed for the NAD conformation at the LQTS locus. Additionally, inverse significant differences were detected for the indel percentage for the injections with Cas9 mRNA (19.18% ± 2.72 for NAD and 29.82% ± 3.14 for TAP, p-value 0.01).

In previous publications, the distance between the intended mutation and the sgRNA cut site has been shown to affect the editing efficiency^21^. To characterize this effect in the context of the *cacna1c* gene, we applied the approach described by Boel et al.^10^. The incorporation of the KI would only generate a single mismatch at the sgRNA binding site for both loci. To avoid re-cutting of the integrated repair sequence, additional synonymous mutations were integrated in the sgRNA binding domain of the ssODN. The effect of the distance between the KI site and the CRISPR-Cas9 cut site on the KI efficiency was then determined based on the rate of incorporation of these different synonymous mutations. The synonymous mutations incorporated at the LQTS locus were at 1, 3 and 6 BP from the sgRNA cut site, with the KI site at 5 BP. For the BrS locus, synonymous mutations at 1 and 12 BP were used, the KI site was at 8 BP.

**Figure 3:**
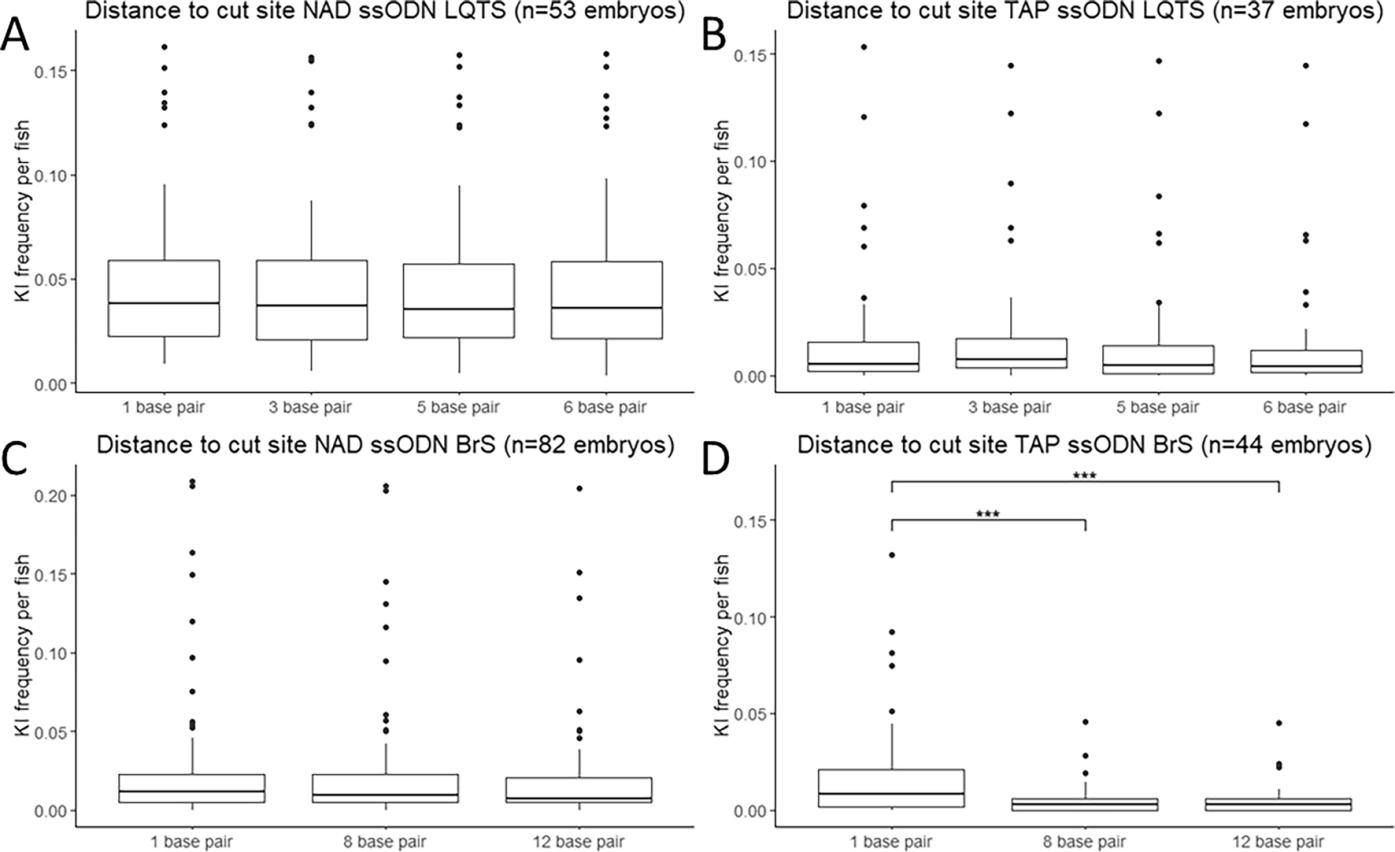
Effect of distance to the cut site on KI efficiency. (**A**) Frequency of KI per embryo for the non-target asymmetric PAM distal ssODN conformation for the LQTS locus (n = 37). (**B**) Frequency of KI per embryo for the target asymmetric PAM proximal ssODN conformation for the LQTS locus (n = 53). (**C**) Frequency of KI per embryo for the non-target asymmetric PAM distal ssODN conformation for the BrS locus (n = 82). (**D**) Frequency of KI per embryo for the target asymmetric PAM proximal ssODN conformation for the BrS locus (n = 44). Statistical testing was performed with negative binomial regression with use of the Tukey’s HSD (honestly significant difference) test for multiple correction, not significant results are not displayed, ***p-value < 0.001, **p-value < 0.01, *p-value < 0.05. *KI* knock-in, *NAD* non-target asymmetric PAM distal, *TAP* target asymmetric PAM proximal.

For the LQTS locus, the average rate of incorporation was very similar for both conformations, ranging from 5.14% (at 5–6 BP) to 5.42% (at 1 BP) for the NAD conformation (n = 37, Fig. 3A) and from 1.36% at 6 BP to 1.72% at 3 BP for the TAP conformation (n = 53, Fig. 3B). For the BrS locus, no significant differences were detected for the NAD conformation (editing rate ranging from 1.95% for 12 BP to 2.56% at 1 BP (n = 82, Fig. 3C). However, significantly higher efficiencies were detected for the TAP conformation at 1 BP to the cut site (1.87%), compared to 8 BP (0.52%, p-value < 0.001) and 12 BP (0.47%, p-value < 0.001, n = 44, Fig. 3D). The injections were performed with Cas9 protein but we also observed similar patterns for Cas9 mRNA (Supplementary Fig. S2).

### Embryo selection with the zebrafish embryo genotyper (ZEG)

**Figure 4:**
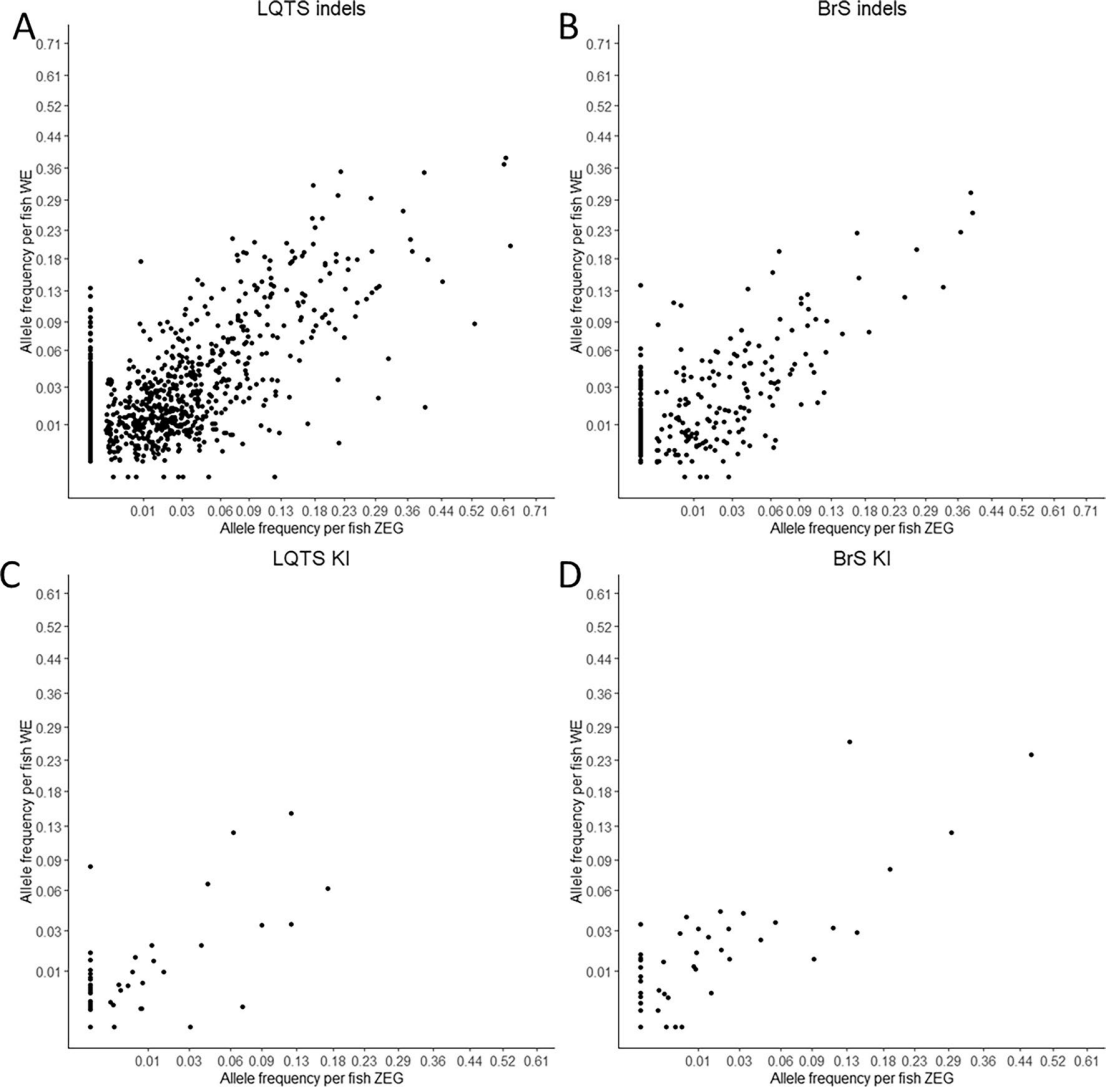
Correlation between zebrafish embryo genotyper (ZEG) and whole embryo (WE) samples. Correlation of indel (**A**) and knock-in (**B**) somatic editing frequency for the LQTS locus with square root transformation of the x- and y-axes. Correlation of indel (**C**) and knock-in (**D**) somatic editing frequencies for the BrS locus. *KI* knock-in, *indel* insertion/deletions.

To further improve KI efficiency, we investigated whether the use of the ZEG would enable an early selection of embryos with the highest KI rates (see Supplementary Fig. S3 for experimental design and rationale). These experiments were performed with the TAP conformation for the LQTS locus and the NAD conformation for the BrS locus. All injections were performed with Cas9 protein. To assess the accuracy of the ZEG, we performed NGS analysis on individual embryos, which were genotyped with the ZEG and subsequently sacrificed for DNA extraction by whole embryo lysis. To obtain a broader overview on the performance of the ZEG for different editing efficiencies, we compared the ZEG estimates for individual indels as well as KIs. We selected the 10 most frequent indels for each locus for this analysis (Supplementary Tables S1, S2). For the LQTS locus, we observed a strong correlation between the indel rates (Pearson’s r = 0.76, p < 0.001, n = 127, Fig. 4A) and a moderate correlation between the KI rates (Pearson’s r = 0.64, p < 0.001, n = 51, injected with TAP ssODN conformation, Fig. 4B). For the BrS locus, the correlation was very strong for the indel rates (Pearson’s r = 0.83, p < 0.001, n = 43, Fig. 4C) and strong for the KI rates (Pearson’s r = 0.69, p < 0.001, n = 43, injected with NAD ssODN conformation, Fig. 4D).

To further evaluate the performance of the ZEG as a selection tool, we compared the average whole embryo somatic editing frequencies of embryos selected by the ZEG, at thresholds of 2, 5 and 10%, to the excluded embryos with negative binomial regression (Tables 1, 2). These selection thresholds were chosen empirically based on our observation of low yield with selection thresholds < 2% and too few (or no) selected fish with selection thresholds > 10%, especially for alleles with lower somatic editing efficiencies.

**Table 1:** Zebrafish embryo genotyper-based embryo selection at the LQTS locus (n = 51 fish for KI and n = 127 fish for indels).

Editing outcome	N alleles in model	Selection threshold (%)	Average SEF—all fish (%)	Average SEF—excluded fish (%)	Average SEF—selected fish (%)	Fold increase	Significance
KI	1	2	1.52	0.69	5.42	2.56	***
All indels	10	2	2.83	1.29	6.32	1.23	***
Indels < 1%	3	2	0.76	0.63	1.92	1.53	***
Indels 1–5%	6	2	2.11	1.57	3.32	0.58	***
Indels > 5%	1	2	13.38	8.67	13.82	0.03	*
KI	1	5	1.52	0.83	6.67	3.38	**
All indels	10	5	2.83	1.57	9.55	2.38	***
Indels < 1%	3	5	0.76	0.68	3.79	3.99	***
Indels 1–5%	6	5	2.11	1.80	4.43	1.10	***
Indels > 5%	1	5	13.38	8.62	14.49	0.08	***
KI	1	10	1.52	1.11	8.09	4.32	NS
All indels	1	10	2.83	1.95	12.92	3.57	***
Indels < 1%	3	10	0.76	0.76	1.40	0.84	NS
Indels 1–5%	6	10	2.11	2.00	5.07	1.41	***
Indels > 5%	1	10	13.38	9.73	15.99	0.20	***

*KI* knock-in, *indel* insertion/deletion, *SEF* somatic editing frequency (according to whole embryo samples), *N* number, *NS* not significant.

*p-value < 0.05, **p-value < 0.01, ***p-value < 0.001 (based on negative binomial regression with selected and excluded as fixed factors and indel sequence as random factor if > 1 indels were considered in the analysis). The fold increase was calculated as (Average SEF selected fish – average SEF all fish)/average SEF all fish.

**Table 2:** Zebrafish embryo genotyper-based embryo selection at the BrS locus (n = 43 fish for KI and n = 43 fish for indels).

Editing outcome	N alleles in model	Selection threshold (%)	Average SEF—all fish (%)	Average SEF—excluded fish (%)	Average SEF—selected fish (%)	Fold increase	Significance
KI	1	2	3.02	1.19	7.73	1.56	***
All indels	10	2	1.93	0.88	5.85	2.03	***
Indels < 1%	5	2	0.64	0.48	3.60	4.62	***
Indels 1–5%	3	2	1.38	0.93	3.01	1.18	***
Indels > 5%	2	2	5.96	3.08	7.85	0.32	***
KI	1	5	3.02	1.38	10.20	2.38	***
All indels	10	5	1.93	1.14	8.37	3.34	***
Indels < 1%	5	5	0.64	0.48	7.45	10.62	***
Indels 1–5%	3	5	1.38	1.07	6.17	3.46	***
Indels > 5%	2	5	5.96	3.96	9.03	0.51	***
KI	1	10	3.02	1.44	12.77	3.23	***
All indels	10	10	1.93	1.46	12.56	5.52	***
Indels < 1%	5	10	0.64	0.49	11.51	16.96	***
Indels 1–5%	3	10	1.38	1.14	11.74	7.49	***
Indels > 5%	2	10	5.96	4.82	13.03	1.19	***

*KI* knock-in, *indel* insertion/deletion, *SEF* somatic editing frequency, *N* number, *NS* not significant.

***p-value < 0.001 (based on negative binomial regression with selected and excluded as fixed factors and indel sequence as random factor if > 1 indels were considered in the analysis). The fold increase was calculated as (Average SEF selected fish – average SEF all fish)/average SEF all fish.

To obtain more representative samples for this analysis, the indels were grouped based on their average somatic editing frequency (< 1%, 1–5% and > 5%) prior to the selection. For groups of more than one indel, the individual indels were included in the model as random intercepts. The details on the performance of the ZEG with all indel alleles and the absolute numbers of selected and excluded fish are summarized in Supplementary Tables S1 and S2.

**Figure 5:**
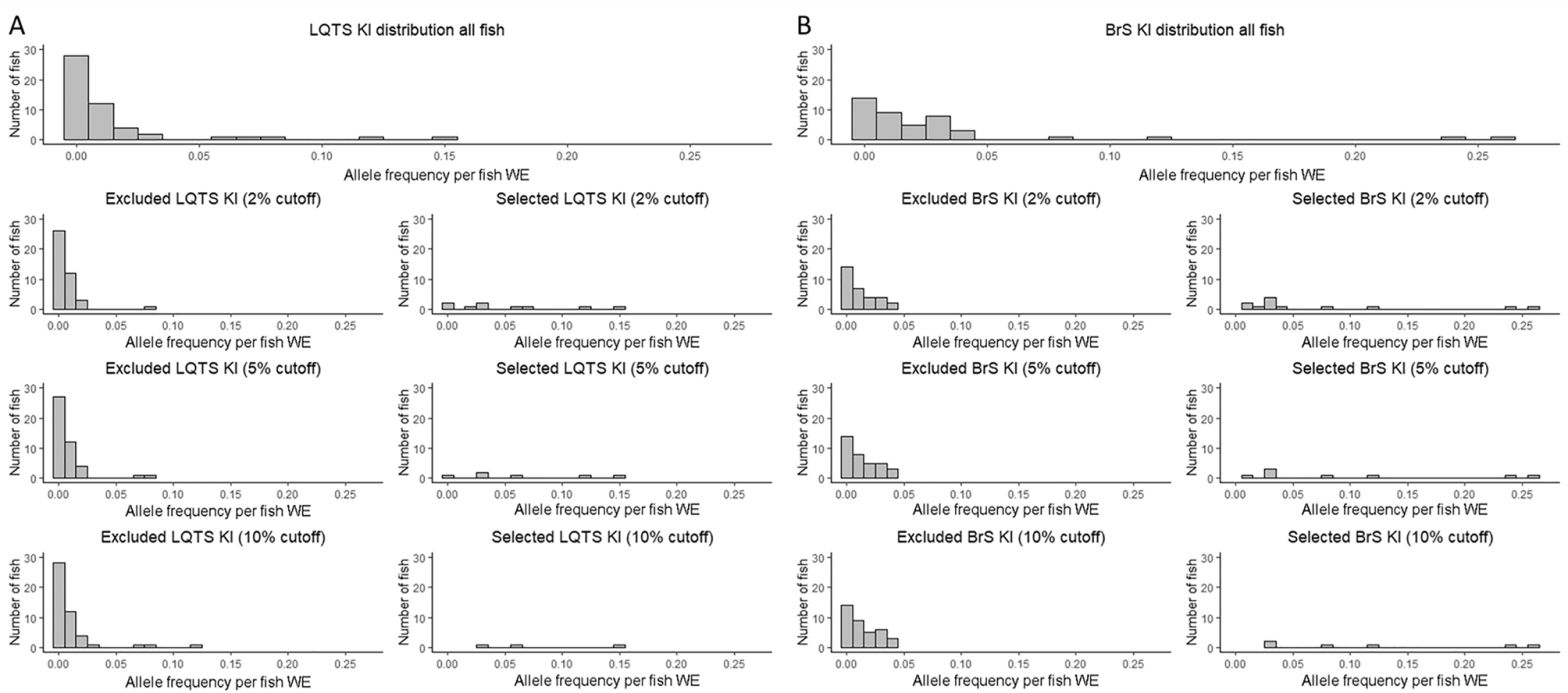
Zebrafish embryo genotyper-based knock-in selection. Histogram of distributions of KI counts of all fish (before selection) and excluded and selected fish after selection with ZEG at 2%, 5% and 10% threshold at the LQTS (n = 51) (**A**) and BrS (n = 43) (**B**) locus. *WE* whole embryo, *KI* knock-in, *indel* insertion/deletion.

For the LQTS locus, we observed a significant distinction between the somatic editing frequency of the excluded and selected fish for all editing outcomes at thresholds of 2% and 5% (Table 1). No significance was observed for the KI and the “indels < 1%” groups at a 10% threshold, which suggests the ZEG is less performant for low-frequency alleles at high selection thresholds at this locus. Additionally, although higher selection thresholds led to a higher average somatic editing efficiency, some fish with high somatic editing frequencies were wrongly excluded (Fig. 5A) and the number of selected fish was low (Supplementary Tables S1, S2). The ZEG performed very well for the selection of the KI at the 2% and 5% selection thresholds, with, respectively, a 3.38 and 4.32-fold increase in the selected group compared to the overall somatic editing efficiency (Table 1).

For the BrS locus, we observed highly significant distinctions between the somatic editing frequency of the excluded and selected fish for all editing outcomes at all thresholds (Table 2). Contrary to the LQTS locus, an especially high fold increase was obtained for the low frequency (< 1%) indels across all selection thresholds, with a 16.96-fold increase for the > 10% cutoff, representing a rise from a somatic editing frequency of 0.64% prior to selection, to 11.51% after selection (Table 2). For the KI, a fold increase of 2.56, 3.38 and 4.32 was obtained for thresholds at > 2%, > 5% and > 10%, respectively. We also observed a high sensitivity for the BrS assay with few excluded fish with high KI frequencies, even at selection thresholds of > 5% and > 10% (Fig. 5B).

### Germline transmission

To assess the germline transmission of the KI allele after ZEG selection, we raised embryos to adulthood and genotyped their offspring. For the LQTS locus, we initially raised several batches of embryos injected with Cas9 mRNA combined with a TAP ssODN, selected at a very low threshold (> 0.02%). We only observed a single fish with germline transmission of the KI allele in the group with the highest somatic editing efficiency (Table 3). We raised several additional batches with a higher selection threshold (> 2%), injected with either Cas9 mRNA or protein. None of the four fish in the mRNA group and one of the five fish in the protein group (20%) showed germline transmission of the KI (Table 3). For the BrS locus, a batch raised at a selection threshold of > 2% yielded germline transmission in one out of two fish (50%, Table 3). Expected germline transmission depending on the somatic editing efficiency based on literature are summarized in Supplementary Table S3.

**Table 3:** Germline transmission of the knock-in allele at the *cacna1c* LQTS and BrS loci in selected embryos.

Locus	N fish	Cas9	ssODN	ZEG threshold (%)	Somatic editing efficiency (%)	N fish with KI offspring
LQTS	5	mRNA	120 BP target PAM proximal	0.02	0.33*	0
LQTS	5	mRNA	120 BP target PAM proximal	0.02	2.13*	0
LQTS	10	mRNA	120 BP target PAM proximal	0.02	6.03*	1 (10%)
LQTS	4	mRNA	120 BP target PAM proximal	2	4.40*	0
LQTS	5	Protein	120 BP target PAM proximal	2	4.61*	1 (20%)
BrS	2	Protein	120 BP non-target PAM distal	2	2.9*	1 (50%)

*N* number, *ssODN* single-stranded deoxynucleotide, *ZEG* zebrafish embryo genotyper, *KI* knock-in, *BP* base pair.

*Established from ZEG in embryos selected for raising to adulthood.

## Discussion

Despite the translational advantages of KI models, their application in zebrafish research has been hampered by the difficulties encountered in the generation of genetically edited animals with precise substitutions. Especially in the setting of low KI efficiency, it is necessary, but very cumbersome, to raise, breed and genotype large numbers of fish before a founder with successful germline transmission can be identified. This requires great investments in the number of laboratory animals used, as well as labor, cost and housing space. Improvements in this process are necessary to increase the feasibility of the KI approach. Most previous studies have focused on the optimization of CRISPR components (sgRNA, Cas9 and repair templates) to increase KI rates.

The selection of sgRNAs with a high cutting efficiency is a crucial step in the generation of a KI model^11^. This can be estimated in silico, although even the most recent computational tools show limited predictive accuracy compared to in vitro assays performed on DNA extracted from injected zebrafish eggs^22^. Most in vitro assays quantify indels in the extracted DNA by separating PCR amplicons with differing lengths^23,24^ or extracting underlying indel traces from Sanger chromatograms^25,26^.

We compared examples of both techniques (CRISPR-STAT and ICE, respectively) to the results of NGS sequencing data, which is considered the gold standard. Our findings show that both tools perform well, although a trend to underestimate the actual indel percentage was observed. This finding corresponds with previous literature, where ICE was found to underestimate the presence of edited alleles^22^. Additionally, the performance of the CRISPR-STAT analysis appeared more dependent on the types of indels occurring at the sgRNA cut site, with lower detection rates for small (1–2 BP) indels.

Both Cas9 protein and mRNA have successfully been used in the past to generate zebrafish KI lines^14,24,27,28^. Despite similar somatic editing efficiencies, higher rates of germline transmission have been observed for protein compared to mRNA^12^. In our study, we indeed confirmed overall higher indel and KI rates in embryos injected with Cas9 protein compared to mRNA, at both the LQTS and BrS locus. Although additional comparisons at different loci are required, both our own findings and previous literature^14,15^ appear to further confirm improved germline transmission with Cas9 protein.

As Cas9 protein is active immediately after injection, it generates double stranded breaks at an earlier developmental stage, compared to Cas9 mRNA, which first needs to undergo translation. This difference in timing may contribute to the improvement of the indel and KI rates, as well as the increased germline transmission we observed by use of the protein. Specifically for our approach, the use of the protein may also increase the accuracy of the ZEG selection, as previous studies have shown higher correlations between somatic tissue (fin clips) in adult founder fish and the chance of germline transmission^12^.

The conformation of the repair template used for HDR has proven to be an important determinant of KI efficiency^10,11^. The most frequently used conformations mainly differ in length, complementarity and the use of either single or double stranded DNA. Successful HDR has been described previously with (double stranded) plasmids with large (400–500 BP) homology arms at both sides of the intended mutation^12,29,30^. Nonetheless, this approach requires a plasmid containing the zebrafish genomic sequence as well as the intended mutation, which can be difficult to generate.

The ssODN approach has the advantage of being easier to implement, as the production of ssODNs requires less effort^10^. Additionally, ssODNs carry less risk of random integration in the genome compared to double stranded templates^31^. The length, symmetry (length of template arms on both sides of the sgRNA cut site) and complementarity of the ssODN repair template appears to significantly contribute to HDR success rates^10,14^. A comparison across four different loci identified optimal KI generation for TAP and NAD conformations with a length of 120 BP^10^. Despite the worse performance of shorter symmetric repair templates compared to other ssODN conformations^10^ and plasmids^12^, symmetric ssODNs of 50 BP have also been used to successfully generate KI lines at several loci^24^.

In our hands, the 120 BP NAD conformation injected with Cas9 protein clearly outperformed the TAP conformation, as well as all injections with Cas9 mRNA, at both loci (with caution of possible injection-related bias at the LQTS locus, as determined by higher indel rates in the NAD ssODN-Cas9 protein group). For future experiments, we would recommend prior testing of the NAD and TAP conformations to determine the optimal gene- or site-specific conditions.

Although the distance of the intended mutation to the sgRNA cut site is an important determinant of KI efficiency in cellular models^21^, in zebrafish this effect appears to be variable^10^. In our study, we found a significant increase in KI rates for synonymous mutations at 1 BP from the cut site specifically for the TAP conformation at the BrS locus. As these findings were incongruent across the two targeted loci, it is possible that the degree of dependence on the distance to the cut site may not be gene specific, and rather depend on the ssODN used and the genomic context. The finding of consistent editing rates at distances up to 8–12 BP from the cut site for the other conditions is particularly relevant, as greater flexibility in the distance to the cut site will broaden the editing window and increase the number of candidate sgRNAs for the generation of specific mutations.

Several studies have also looked at the addition of HDR stimulating compounds to increase editing efficiency, with variable success^10,12,13,15,32^. Reference^12^ describe a 2.6-fold increase of the percentage of embryos with any KI events (as detected by allele-specific PCR) by administration of SCR7, a NHEJ inhibitor. The same compound, as well as other tested NHEJ blockers (NU7441, KU0060648) and HDR stimulators (RS1, L755507), resulted in no significant improvement of somatic editing efficiency (as detected by NGS) in Boel et al.^10^. De Vrieze et al.^15^ observed that morpholino-induced knockdown of the *xrcc6* gene, which encodes a critical component of the NHEJ pathway, has variable and site-dependent effects on the KI efficiency.

Despite these extensive efforts, the selection of optimal KI conditions for individual experiments remains challenging. Methodological differences hamper a consistent extrapolation, as it is difficult to compare experiments with different KI detection methods. Moreover, sgRNA- or locus-specific effects, such as chromatin accessibility, could further confound the KI efficiency interpretation^13^. As a more generalizable approach, pre-selection of adult fish with somatic editing demonstrated on fin clip tissue has been shown to increase the chance of identifying a progenitor with stable germline transmission for Cas9 protein injected zebrafish^12^. In our study, we extend this approach by expediting the selection process to the embryonal stage with the application of the ZEG.

Although other minimally-invasive early genotyping methods have been described^33–36^, the ZEG is commercially available and it has the advantage of enabling relatively easy high-throughput genotyping with minimal lethality at 72 h post-fertilization^17,18^. By performing NGS-based allele detection we were able to show a moderate to very strong correlation between the KI and indel rates detected by the ZEG compared to effective KI and indel rates in embryos for the LQTS and BrS locus.

We established that the ZEG is particularly efficient at the BrS locus, with an almost 17-fold increase in the somatic editing efficiency after selection and a higher specificity of the assay at the BrS locus, especially at higher selection thresholds. The differences observed between the LQTS and BrS locus are likely caused by the varying library prep protocols, which include prior PCR amplification for the LQTS locus. It is likely that immediate amplification with molecular identifiers and barcodes at the BrS locus increased the accuracy of the technique and we would recommend this approach in the future, if technically feasible.

Performing the genotyping at the larval stage enables a more high-throughput workflow and reduces the number of adult fish required for downstream selection. As the ZEG can efficiently process large numbers of embryos, it will likely be particularly useful for experiments with low initial KI rates, as it will improve the germline transmission by enriching the raised fish for higher KI efficiencies. Indeed, in our experiment we observed a higher fold increase of the somatic editing efficiency for less frequent indels, especially for the BrS locus.

Despite our initial promising results, further experiments will be needed to expand on the relationship between the selection threshold, somatic editing efficiency and germline transmission. Despite reaching a somatic editing efficiency of 6.03% in a batch injected with Cas9 mRNA (Table 3), we only observed germline transmission in one out of the 10 fish (10%) who reached sexual maturity. Transmission rates of up to 9% were previously already observed with a much lower somatic efficiency of 1.92% (Supplementary Table S3)^14^. However, the value we observed was highly influenced by a single outlier with a KI allele frequency of 65% (as the embryos are pooled after selection, it is unknown if this embryo survived up to adulthood). When this outlier is no longer considered, the editing efficiency drops to 2.18%. The germline transmission of 20% in Cas9 protein injected fish with a somatic editing efficiency of 4.61% was comparable to previously published results with similar somatic editing efficiencies (30% transmission with somatic editing efficiency of 3.4%, Supplementary Table S3)^15^. We also observed a high percentage of germline transmission in the group selected for the BrS locus (somatic editing efficiency of 2.9% at time of selection), with one out of the two surviving fish transmitting the KI.

For our loci, raising representative sample sizes of fish selected at different thresholds was extremely difficult, as only a small number of fish survived to breeding age, especially after injections with Cas9 protein. Embryonic lethality was previously observed for homozygous nonsense mutations in *cacna1c* (forward mutagenesis screens derived *isl* mutants)^37^. As the deaths also occurred in non-dysmorphic larvae who did not undergo the ZEG selection procedure at an age of > 5 days post-fertilization, it seems likely that they were related to biallelic loss of *cacna1c*, rather than ZEG- or injection-related toxicity.

Our workflow requires the immediate availability of NGS, as the results are needed for embryo selection before 5 days post-fertilization. We realize that the high cost and lack of access to NGS infrastructure could present a limitation to the implementation of our protocols in other labs. Although we considered the use of NGS as essential to the validation of the data obtained by the ZEG, follow-up studies could explore the performance of the ZEG with other methods for KI genotyping in founder fish (e.g. restriction enzyme digest specific for the intended KI, allele-specific PCR^12^ or Sanger sequencing^25^). However, it is unlikely that other methods would yield equally accurate results as obtained by NGS. Additionally, it would be interesting to study whether combining different early genotyping methods (e.g. ZEG and proteinase treatments)^36^ could lead to further improvements.

Overall, our findings show that the ZEG is a promising tool for improvement of KI efficiency. Additionally, our method is generalizable to different loci and has proven to be particularly beneficial for low somatic editing efficiencies. We expect that the application of this technique will facilitate the generation of KI models and reduce the number of animals required for this purpose.

## Methods

### Design and production of sgRNAs and Cas9

Single guide RNAs (sgRNAs) were designed with CHOPCHOP^38^ and CRISPOR^39^. For the LQTS locus, only sgRNAs which conformed to the 5′ sequence requirement of an in vitro transcription promoter (GG- for T7 and GA- for SP6) were considered. sgRNAs with cut sites closest to the mutations were generated by in vitro transcription (LQTS locus) or ordered from Synthego (BrS locus) (modified sgRNA, CRISPRevolution sgRNA EZ Kit) (Supplementary Table S4).

For the LQTS locus, the custom sgRNA target sequence coupled to the required promoter was ordered from IDT and annealed with Phusion High-Fidelity DNA Polymerase (Bioké) to a universal DNA strand containing the invariant components of the sgRNA (IDT)^40^. This construct formed the template used for the in vitro transcription reaction with the T7 High Yield RNA Synthesis Kit (Bioké). The incubation step of the transcription reaction was performed overnight to increase yield. RNA purification was performed by sodium-acetate precipitation^40^. Successful transcription was confirmed by gel electrophoresis.

Cas9 mRNA was generated from the pCS2-nCas9n (Addgene #47929) plasmid after linearization with NotI-HF (Bioké) by in vitro transcription with the mMESSAGE mMACHINE SP6 Transcription Kit with a 2 h incubation step. Purification was performed by LiCl precipitation, available from the transcription kit. Cas9 protein was ordered from Bioké. All RNA constructs were stored at − 80 °C.

### Repair template design

The ssODN conformations have a length of 120 BP and contain a longer (90 BP) sequence on either the PAM-proximal (TAP) or the PAM-distal side (NAD). The length of long arm of the TAP ssODN for the LQTS locus was slightly altered (shortened by 9 BP) in order to avoid a single nucleotide polymorphism (SNP) occurring at this site (Supplementary Table S4). The total length of the ssODN remained unchanged. The target conformation is based on the DNA-strand which binds the sgRNA, with the non-target as its reverse complement. Additional synonymous mutations within the sgRNA binding sequence were incorporated in each ssODN to reduce the risk of re-cutting by Cas9 after successful incorporation of the repair sequence. The synonymous mutations were located at 1, 3 and 6 BP from the cut site at the LQTS locus and 1 and 12 BP at the BrS locus (Supplementary Table S4). The ssODNs were ordered as ultramer oligonucleotides, without PAGE purification (IDT, 4 nmol) for the LQTS locus and as Alt-R HDR Donor oligos (IDT) for the BrS locus.

### Zebrafish husbandry, CRISPR-Cas9 injections and DNA extraction

Approval for this study was obtained from the Ethical Committee for Animal Testing of the University of Antwerp. Zebrafish were handled and maintained according to standard practice^41^. All experiments were performed in accordance with the ARRIVE guidelines and the Directive 2010/63/EU. The experiments were performed with tg(*myl7:Ace2N-mNeon/R-GECO*) zebrafish, a transgenic line with cardiac expression of genetically encoded fluorescent voltage and calcium reporters, generated at our lab from the AB strain.

Zebrafish embryos were injected intracellularly with 200 pg Cas9 RNA, 50 pg sgRNA and 50 pg ssODN or 250 pg Cas9 protein, 25 pg (LQTS locus) or 50 pg (BrS locus) sgRNA and 50 pg ssODN at the 1-cell stage. The injection mix was supplemented by 10% Phenol Red to improve visibility. Non-dysmorphic embryos were selected for genotyping by whole embryo lysis at 1–3 days post-fertilization, with the sodium hydroxide extraction procedure^40^ and/or by the ZEG according to the manufacturers’ protocol^18^ at 3 days post-fertilization. Briefly, 24 individual embryos (suspended in 12 µl drops of embryo medium for each embryo) were transferred to circular roughened areas of a ZEG chip and shaken for 7.5 min in the ZEG base unit. This procedure leads to shedding of, on average, 21 surface cells of each embryo. 10 µl of the fluid surrounding the embryo is extracted and used for downstream analysis, while the embryos are transferred to individual wells in a 24-well plate. 1 µl of whole embryo and 5 µl of ZEG DNA was used for all downstream experiments.

### Insertion/deletion frequency estimation

Indel frequency estimation was performed for individual embryos on genomic material obtained from whole embryo lysis. To assess the cutting efficiency of the selected sgRNAs, we evaluated the performance of two tools used for indel frequency estimation: CRISPR-STAT and ICE^23,25^. CRISPR-STAT relies on a high-resolution microcapillary electrophoresis for the detection of small differences in amplicon sizes between fluorescently labelled PCR products (Fig. 1A). The ICE CRISPR Analysis Tool reconstructs indel traces from Sanger sequencing chromatograms (Fig. 1B).

For CRISPR-STAT, primers were designed to amplify a region of approximately 200–250 BP with the CRISPR-Cas9 cut site roughly in the middle and labeled with 6-fluorescein amidite (FAM, IDT) (Supplementary Table S4). PCR products were analyzed on an ABI3130XL (Applied Biosystems) in the presence of an internal sizing standard (ROX). Amplicon sizes and peak intensities were determined using ABI GeneMapper software v3.7 (Applied Biosystems)^23^. To obtain an indel percentage for each sample, the intensity of all indel peaks was summed and divided by the sum of all peaks (indel and wildtype). ICE^25^ was used to extract editing outcomes from Sanger sequencing data (Supplementary Table S4).

### NGS library prep

For the LQTS locus, the genomic DNA extracted by the ZEG was PCR-amplified and diluted 1:100 prior to the NGS library prep, as we were unable to successfully amplify the genomic DNA with primers containing overhangs otherwise. The PCR conditions were optimized for each locus to obtain successful amplification with minimal primer dimer formation (Supplementary Table S4).

Primers were designed to amplify a region of approximately 200–250 BP, with the CRISPR-Cas9 cut site roughly in the middle. Overhangs containing unique molecular identifiers (UMI) and binding sites for paired-end sequencing NGS primers were added to the primers. These primers were used to amplify each sample individually in the first PCR step, with 35 amplification cycles for the LQTS protocol and 28 cycles for the BrS protocol. In the second PCR step, overhang primers containing unique sample identifiers for each embryo and flow cell binding sites were added in 12 cycles to the PCR product of step 1, which was 1:10 diluted for the LQTS locus and undiluted for the BrS locus. The concentration of the resulting PCR products was determined with the Qubit dsDNA HS (High Sensitivity) Assay Kit (ThermoFisher Scientific), with simultaneous quantification for all the samples by use of the VICTOR Multilabel Plate Reader (PerkinElmer). Equal amounts of all samples were pooled (in our hands pools of > 300 embryos were feasible), purified with magnetic beads, diluted to a concentration of 10 pM and spiked with 30% PhiX Control v3 Library (Illumina). Paired-end, dual-indexed sequencing with 2 × 150 BP cycles was performed on a MiSeq instrument (Illumina).

### Analysis of NGS data

NGS data analysis was performed using the previously published BATCH-GE script^16^. The script was adjusted by replacing the Picard Tools duplicate read removal by UMI-tools, as Picard Tools proved too stringent for targeted amplification (the adjusted scripts can be accessed at the following repositories: https://bitbucket.org/tychoCanterCremers/smip_pipeline/src/master/ and https://bitbucket.org/tychoCanterCremers/batch-ge_pipeline/src/master/). For the insertions and deletions, individual editing outcomes were adapted in Pycharm for downstream analysis (the scripts used can be accessed at the following repository: https://bitbucket.org/tychoCanterCremers/batch-ge_post_analysis/src/master/). All samples with a total number of NGS reads below 100 were excluded from the analysis. The NGS pipeline was validated on not injected control embryos and showed a low average indel percentage (0.07% for the LQTS locus and 0.06% for the BrS locus), an absence of KIs for the LQTS locus and a low average KI percentage for the BrS locus (0.11%) in whole embryo lysis samples (similar rates were observed for ZEG samples, Supplementary Table S5).

### KI assessment in F1 generation

The presence of the desired KI in genomic DNA was assessed by restriction enzyme (RE) digest or Sanger sequencing for the LQTS locus and Sanger sequencing only for the BrS locus. RE digest was performed by PCR amplification of the region of interest, followed by incubation at 37 °C with Hpy99I (Bioké). Hpy99I cuts DNA only in the presence of the desired KI, which can be observed by the appearance of additional bands on gel electrophoresis. Positive RE digest samples were subsequently verified by Sanger sequencing.

### Statistical analysis

The average allele frequency was calculated by averaging the allele frequencies across all embryos included in the analysis. Correlation and negative binomial regression were performed in R. To analyse the correlation between the whole embryo lysis- and ZEG-derived read counts of KI or indel events, we used the absolute numbers of the NGS reads containing the correct KI or indel for each embryo, with normalization of the whole embryo samples to the total read counts in ZEG samples. For the negative binomial regression, the absolute counts of KI reads of whole embryo samples were used and the total read count per sample was added to the model as a logarithmic offset. Tukey’s HSD (honestly significant difference) test (ssOND and distance to cut site comparisons) or Bonferroni correction (ZEG selection) were applied to correct for multiple testing. Graphics were generated in R and Tableau.

## Supplementary Information

Supplementary Information.

## Data Availability

All datasets and scripts used and/or analyzed during the current study are available from the corresponding author on reasonable request. The raw sequencing data has been made available at the European Nucleotide Database via the project Accession Number PRJEB56568.
