# Supplementary material for: Improved selection of zebrafish CRISPR editing by early next-generation sequencing based genotyping
Source: Sci Rep. 2023 Jan 27;13:1491. doi: 10.1038/s41598-023-27503-9 (PMC9883431; doi:10.1038/s41598-023-27503-9)
Supplement: Supplementary file 1 — Supplementary Information. [file 41598_2023_27503_MOESM1_ESM.pdf]

Improved selection of zebrafish CRISPR editing by early next-generation sequencing based genotyping

Ewa Sieliwonczyk, Bert Vandendriessche, Charlotte Claes, Evy Mayeur, Maaike Alaerts, Philip Holmgren, Tycho Canter Cremers, Dirk Snyders, Bart Loeys, Dorien Schepers

Supplemental data:

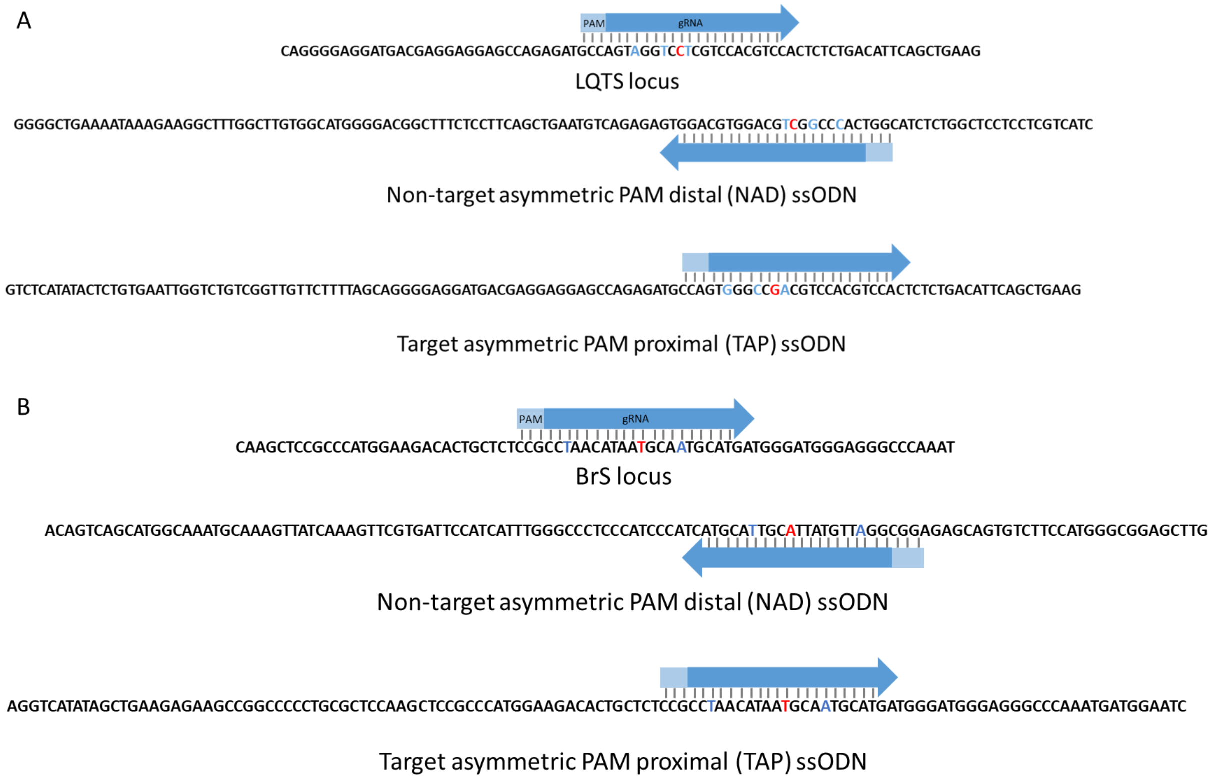

Fig. S1: Single stranded deoxynucleotide (ssODN) design for the LQTS locus (A) and the BrS locus (B). Red: intended knock-in mutation, blue: synonymous mutations, PAM: protospacer adjacent motif

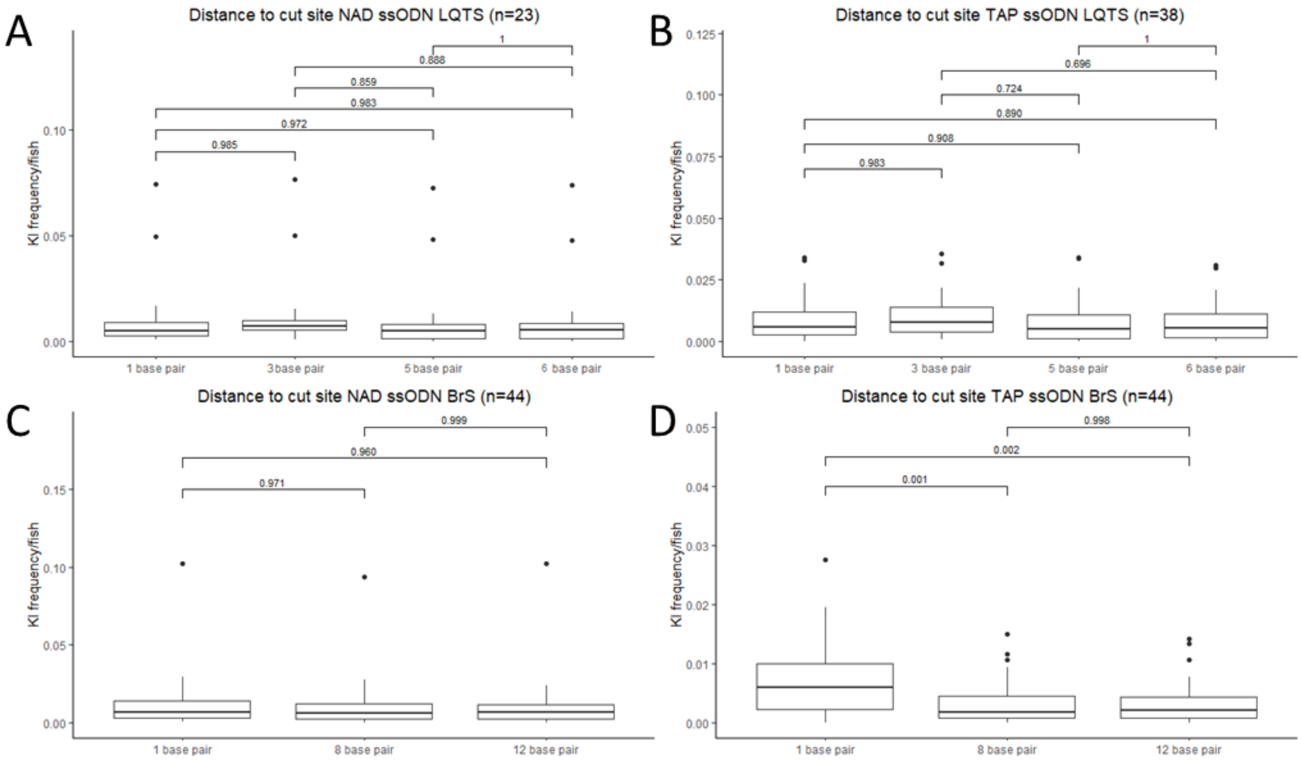

Fig. S2: effect of distance to the cut site on KI efficiency with Cas9 mRNA. 3A: percentage of KI per embryo for the non-target asymmetric PAM distal ssODN conformation for the LQTS locus (n = 23). 3B: percentage of KI per embryo for the target asymmetric PAM proximal ssODN conformation for the LQTS locus (n = 38). 3C: percentage of KI per embryo for the non-target asymmetric PAM distal ssODN conformation for the BrS locus (n = 44). 3D: percentage of KI per embryo for the target asymmetric PAM proximal ssODN conformation for the BrS locus (n = 44). KI: knock-in, NAD: non-target asymmetric PAM distal, TAP: target asymmetric PAM proximal

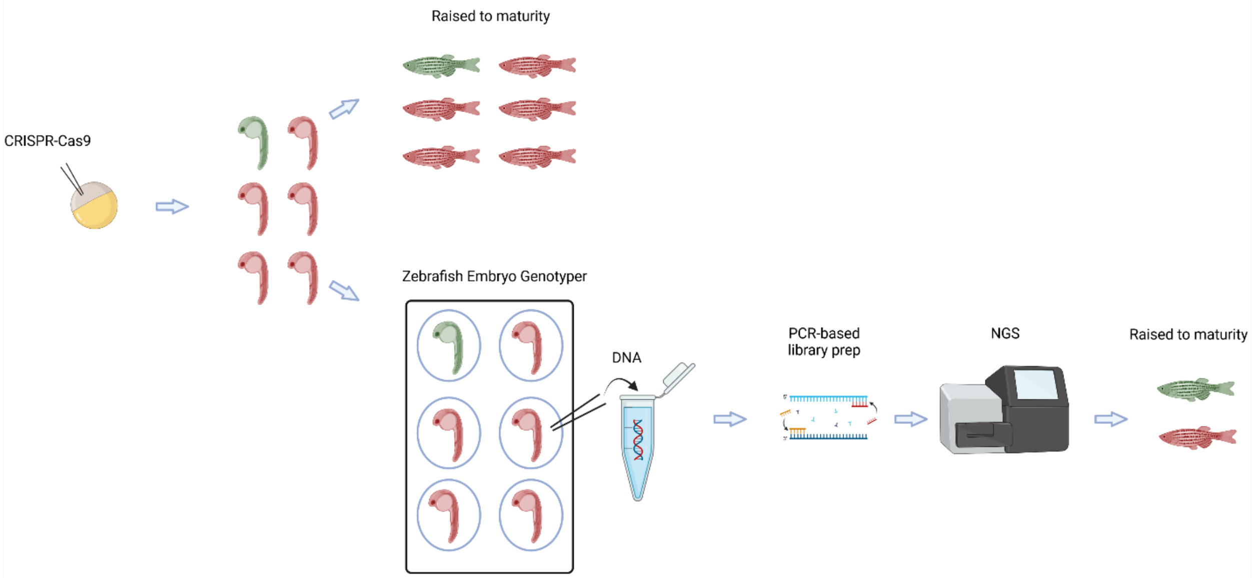

Fig. S3: Rationale and experimental design of Zebrafish Embryo Genotyper selection procedure. Red fish: no germline transmission of the KI. Green fish: germline transmission of the KI.

Sequence	Mean SEF (%)	Mean SEF - unselected (%)	Mean SEF - selected (%)	N unselected (% of total)	N selected (% of total)	Selection threshold (%)
DEL4GATGCCAGTA[GGTC]CTCGTCCACG	0,57	0,52	1,33	119 (94)	8 (6)	2
DEL4GATGCCAGTA[GGTC]CTCGTCCACG	0,57	0,54	2,60	125 (98)	2 (2)	5
DEL4GATGCCAGTA[GGTC]CTCGTCCACG	0,57	0,57	0,97	126 (99)	1 (1)	10
DEL8AGCCAGAGAT[GCCAGTAG]GTCCTCGTCC	0,74	0,53	2,46	113 (89)	14 (11)	2
DEL8AGCCAGAGAT[GCCAGTAG]GTCCTCGTCC	0,74	0,57	5,01	122 (96)	5 (4)	5
DEL8AGCCAGAGAT[GCCAGTAG]GTCCTCGTCC	0,74	0,73	1,82	126 (99)	1 (1)	10
DEL1AGATGCCAGT[A]GGTCCTCGTC	0,96	0,84	1,75	110 (87)	17 (13)	2
DEL1AGATGCCAGT[A]GGTCCTCGTC	0,96	0,92	2,54	124 (98)	3 (2)	5
DEL1AGATGCCAGT[A]GGTCCTCGTC	0,96	0,92	0,00	127 (100)	0 (0)	10
DEL10AGAGATGCCA[GTAGGTCCTC]GTCCACGTCC	1,25	1,04	2,13	102 (80)	25 (20)	2
DEL10AGAGATGCCA[GTAGGTCCTC]GTCCACGTCC	1,25	1,03	5,03	120 (94)	7 (6)	5
DEL10AGAGATGCCA[GTAGGTCCTC]GTCCACGTCC	1,25	1,12	18,11	126 (99)	1 (1)	10
DEL3CAGAGATGCC[AGT]AGGTCCTCGT	1,55	1,27	2,29	92 (72)	35 (28)	2
DEL3CAGAGATGCC[AGT]AGGTCCTCGT	1,55	1,37	3,32	115 (91)	12 (9)	5
DEL3CAGAGATGCC[AGT]AGGTCCTCGT	1,55	1,50	2,97	123 (97)	4 (3)	10
INS1GATGCCAGTA[G]GGTCCTCGTC	1,86	1,44	3,03	93 (73)	34 (27)	2
INS1GATGCCAGTA[G]GGTCCTCGTC	1,86	1,54	4,49	113 (89)	14 (11)	5
INS1GATGCCAGTA[G]GGTCCTCGTC	1,86	1,74	4,00	120 (94)	7 (6)	10
DEL9GCCAGAGATG[CCAGTAGGT]CCTCGTCCAC	1,95	1,56	3,19	96 (76)	31 (24)	2
DEL9GCCAGAGATG[CCAGTAGGT]CCTCGTCCAC	1,95	1,90	2,91	120 (94)	7 (6)	5
DEL9GCCAGAGATG[CCAGTAGGT]CCTCGTCCAC	1,95	1,90	0,00	127 (100)	0 (0)	10
DEL1GAGATGCCAG[T]AGGTCCTCGT	2,70	1,91	4,25	84 (66)	43 (34)	2
DEL1GAGATGCCAG[T]AGGTCCTCGT	2,70	2,19	5,27	106 (83)	21 (17)	5
DEL1GAGATGCCAG[T]AGGTCCTCGT	2,70	2,56	5,67	121 (95)	6 (5)	10
DEL1GATGCCAGTA[G]GTCCTCGTCC	3,31	2,65	3,94	62 (49)	65 (51)	2
DEL1GATGCCAGTA[G]GTCCTCGTCC	3,31	3,00	4,48	100 (79)	27 (21)	5
DEL1GATGCCAGTA[G]GTCCTCGTCC	3,31	3,20	4,96	119 (94)	8 (6)	10
DEL4AGAGATGCCA[GTAG]GTCCTCGTCC	13,38	8,67	13,82	11 (9)	116 (91)	2
DEL4AGAGATGCCA[GTAG]GTCCTCGTCC	13,38	8,62	14,49	24 (19)	103 (81)	5
DEL4AGAGATGCCA[GTAG]GTCCTCGTCC	13,38	9,73	15,99	53 (42)	74 (58)	10

Table S1: ZEG performance as selection tool for ten most frequent CRISPR editing outcomes for the LQTS locus. SEF: somatic editing frequency

Sequence	Mean SEF (%)	Mean SEF - unselected (%)	Mean SEF - selected (%)	N unselected (% of total)	N selected (% of total)	Selection threshold (%)
DEL4CTCTCCGCCC[AACA]TAACGCAGTG	0,56	0,38	4,10	41 (95)	2 (5)	2
DEL4CTCTCCGCCC[AACA]TAACGCAGTG	0,56	0,38	4,10	41 (95)	2 (5)	5
DEL4CTCTCCGCCC[AACA]TAACGCAGTG	0,56	0,38	7,88	42 (98)	1 (2)	10
INS2CGCCCAACAT[CA]AACGCAGTGC	0,56	0,04	22,53	42 (98)	1 (2)	2
INS2CGCCCAACAT[CA]AACGCAGTGC	0,56	0,04	22,53	42 (98)	1 (2)	5
INS2CGCCCAACAT[CA]AACGCAGTGC	0,56	0,04	22,53	42 (98)	1 (2)	10
INS2CTCTCCGCCC[AT]AACATAACGC	0,58	0,55	1,20	41 (95)	2 (5)	2
INS2CTCTCCGCCC[AT]AACATAACGC	0,58	0,54	2,40	42 (98)	1 (2)	5
INS2CTCTCCGCCC[AT]AACATAACGC	0,58	0,54	0,00	43 (100)	0 (0)	10
DEL3TGCTCTCCGC[CCA]ACATAACGCA	0,65	0,55	1,90	40 (93)	3 (7)	2
DEL3TGCTCTCCGC[CCA]ACATAACGCA	0,65	0,57	4,10	42 (98)	1 (2)	5
DEL3TGCTCTCCGC[CCA]ACATAACGCA	0,65	0,57	4,10	42 (98)	1 (2)	10
DEL1CTCTCCGCCC[A]ACATAACGCA	0,85	0,89	0,24	40 (93)	3 (7)	2
DEL1CTCTCCGCCC[A]ACATAACGCA	0,85	0,89	0,00	43 (100)	0 (0)	0
DEL1CTCTCCGCCC[A]ACATAACGCA	0,85	0,89	0,00	43 (100)	0 (0)	10
DEL4TGCTCTCCGC[CCAA]CATAACGCAG	1,20	1,04	1,81	34 (79)	9 (21)	2
DEL4TGCTCTCCGC[CCAA]CATAACGCAG	1,20	1,16	1,73	40 (93)	3 (7)	5
DEL4TGCTCTCCGC[CCAA]CATAACGCAG	1,20	1,17	2,65	42 (98)	1 (2)	10
DEL10AGACACTGCT[CTCCGCCCAA]CATAACGCAG	1,29	0,52	6,05	37 (86)	6 (14)	2
DEL10AGACACTGCT[CTCCGCCCAA]CATAACGCAG	1,29	0,53	16,95	41 (95)	2 (5)	5
DEL10AGACACTGCT[CTCCGCCCAA]CATAACGCAG	1,29	0,59	30,54	42 (98)	1 (2)	10
DEL5CTGCTCTCCG[CCCAA]CATAACGCAG	1,66	1,32	2,44	30 (70)	13 (30)	2
DEL5CTGCTCTCCG[CCCAA]CATAACGCAG	1,66	1,53	3,43	40 (93)	3 (7)	5
DEL5CTGCTCTCCG[CCCAA]CATAACGCAG	1,66	1,65	2,04	42 (98)	1 (2)	10
DEL3GCTCTCCGCC[CAA]CATAACGCAG	5,67	2,79	7,37	16 (37)	27 (63)	2
DEL3GCTCTCCGCC[CAA]CATAACGCAG	5,67	4,22	7,88	26 (60)	17 (40)	5
DEL3GCTCTCCGCC[CAA]CATAACGCAG	5,67	4,61	12,21	37 (86)	6 (14)	10
DEL12CACTGCTCTC[CGCCCAACATAA]CGCAGTGCAT	6,25	3,33	8,36	18 (42)	25 (58)	2
DEL12CACTGCTCTC[CGCCCAACATAA]CGCAGTGCAT	6,25	3,69	10,17	26 (60)	17 (40)	5
DEL12CACTGCTCTC[CGCCCAACATAA]CGCAGTGCAT	6,25	5,02	13,85	37 (86)	6 (14)	10

Table S2: ZEG performance as selection tool for ten most frequent CRISPR editing outcomes for the BrS locus. SEF: somatic editing frequency

Gene - locus	*N fish*	*Cas9*	*ssODN*	*ZEG threshold*	*Somatic editing efficiency (%)*	*N fish with KI offspring*
tp53 - R143H[14]	30	mRNA	126 BP target symmetric	/	0.75*	1 (3%)
tp53 - R143H[14]	41	mRNA	126 BP non-target PAM proximal	/	2*	2 (5%)
tp53 - R217H[14]	38	mRNA	136 BP target symmetric	/	0,2*	0
tp53 - R217H[14]	22	mRNA	126 BP non-target PAM proximal	/	1,92*	2 (9%)
ush2a - C771F[15]	10	Protein	126 BP non-target - PAM proximal	/	3.4*	3 (30%)
ripor2 - del 12[15]	11	Protein	126 BP non-target - PAM proximal	/	8.6*	4 (45%)

Table S3: Germline transmission of the knock-in allele in literature. N: number, ssODN: single-stranded deoxynucleotide, ZEG: zebrafish embryo genotyper, KI: knock-in, BP: base pair, * established from ZEG in embryos selected for raising to adulthood, * established by NGS on pools of embryos from previous injections

	Forward	Reverse
**LQTS ICE primers**	TGGTGGTGGTCAGTGTGTCT	TGCATCTGTGCATCTGAATGT
**LQTS CRISPR-STAT primers**	**CACGACGTTGTAAAACGAC**TGGTCTGTCGGTTGTTCTTTT*	TGACACAAAACCCTGAACCT
**LQTS PCR amplification prior to NGS**	AGCTCTGGGAGCACTCTACG	CATTCTTCACTGGGTCCTCCG
**LQTS NGS primers**	CTCTGTGAATTGGTCTGTCGGT	TGAATACTAAGTGGCACAACCCA
**LQTS sgRNA**	GGACGTGGACGAGGACCTAC**TGG****	
**LQTS NAD ssODN**	GGGGCTGAAAATAAAGAAGGCTTTGGCTTGTGGCATGGGGACGGCTTTCTCCTTCAGCTGAATGTCAGAGAGTGGACGTGGACGTCGGCCCACTGGCATCTCTGGCTCCTCCTCGTCATC***	
**LQTS TAP ssODN**	GTCTCATATACTCTGTGAATTGGTCTGTCGGTTGTTCTTTTAGCAGGGGAGGATGACGAGGAGGAGCCAGAGATGCCAGTGGGCCGACGTCCACGTCCACTCTCTGACATTCAGCTGAAG***	
**BrS ICE primers**	TCAGGACTTCAGCTTTTGCTTCT	GTAGTGTGCCGATACTGGAGC
**BrS CRISPR-STAT primers**	**CACGACGTTGTAAAACGAC**TCAGGACTTCAGCTTTTGCTTCT	GTAGTGTGCCGATACTGGAGC
**BrS NGS primers**	ATTGACCCTGGTGTTTACCC	CTGAAACACAGTCAGCATGGC
**BrS sgRNA**	ATGCACTGCGTTATGTTGGG**CGG****	
**BrS NAD ssODN**	ACAGTCAGCATGGCAAATGCAAAGTTATCAAAGTTCGTGATTCCATCATTTGGGCCCTCCCATCCCATCATGCATTGCATTATGTTAGGCGGAGAGCAGTGTCTTCCATGGGCGGAGCTTG***	
**BrS TAP ssODN**	AGGTCATATAGCTGAAGAGAAGCCGGCCCCCTGCGCTCCAAGCTCCGCCCATGGAAGACACTGCTCTCCGCCTAACATAATGCAATGCATGATGGGATGGGAGGGCCCAAATGATGGAATC***	

Table S4: sgRNA, ssODN and primer sequences. * in bold: M13 sequence, ** in bold: PAM site, *** in red: KI site, in green: synonymous mutations

	LQTS locus		BrS locus	
	**WE**	**ZEG**	**WE**	**ZEG**
**N fish**	20	17	8	8
**N alleles**	22482	18776	9293	8610
**Average indel %**	0,07	0,06	0,06	0,08
**Average KI %**	0	0	0,11	0,19
**Highest indel %**	0,25	0,2	0,52	0,17
**Highest KI %**	0	0	0,17	0,37

Table S5: evaluation of performance of the next generation sequencing pipeline on not injected control embryos. LQTS: long QT syndrome, BrS: Brugada syndrome, WE: samples derived from whole embryo lysis, ZEG: samples derived from the Zebrafish Embryo Genotyper, indel: insertion or deletion, KI: knock in
